# The barriers to offering non-pharmacological pain management as an initial option for laboring women: A review of the literature

**DOI:** 10.18332/ejm/149244

**Published:** 2022-06-10

**Authors:** Matilda A. Ingram, Susannah Brady, Ann S. Peacock

**Affiliations:** 1 School of Nursing, Midwifery and Social Work, University of Queensland, Brisbane, Australia; 2 School of Nursing and Midwifery, University of Newcastle, Newcastle, Australia; 3 College of Healthcare Sciences, James Cook University, Townsville, Australia

**Keywords:** pregnancy, labor, obstetric, midwifery, labor pain, pain management

## Abstract

**INTRODUCTION:**

Many women use pharmacological or non-pharmacological pain management (NPPM) during childbirth, however, evidence shows the usage rates of pharmacological pain management are increasing. The shift towards a biomedical approach to birth care opposes the enduring midwifery philosophy of trusting the woman and her body. Identifying midwives’ beliefs and attitudes towards perceived and actual barriers to offering NPPM as an initial option will provide insight into the factors that affect this.

**METHODS:**

This review of the literature sought to understand midwives’ beliefs and attitudes towards the barriers to offering NPPM as an initial option for laboring women. Peer-reviewed journals were searched for primary research that met the inclusion criteria and explored midwives’ beliefs and attitudes towards the barriers to offering NPPM as an initial option for laboring women. Included studies were evaluated for quality according to the Critical Appraisal Skills Programme (CASP) checklists.

**RESULTS:**

Thirteen qualitative studies met the inclusion criteria and four main themes of barriers to midwives offering NPPM emerged: health system-related, health facility-related, health practitioner-related, and health consumer-related barriers.

**CONCLUSIONS:**

The review of the literature highlighted there are barriers that prevent or delay the initial utilization of non-pharmacological methods of pain management in labor by midwives. These findings can be used as a platform to inform further research into this topic.

## INTRODUCTION

Labor and childbirth are normal physiological processes that are multidimensional and subjective in nature^1^. Over time, midwifery and childbirth has seen a paradigm shift towards a biomedical model of care^2^. The advancements in technology have formed a dichotomy: on one side, there have been reductions in maternal and fetal mortality rates, while on the other, women experience increased rates of intervention which carry further risks^2^. Labor pain management is one avenue of contemporary midwifery that has followed the shift towards this biomedical model of care^3^.

Since the introduction of pharmacological pain management, nitrous oxide gas, opioids and epidural analgesia, have dominated labor pain management^1^. In 2017, 78% of laboring mothers used pharmacological methods of pain management, with 40% using regional analgesia^4^. Non-pharmacological approaches to pain management hold promise in reducing pain and anxiety, and, unlike pharmacological methods, they pose minimal or no risk to the mother and fetus^5^. The utilization of NPPM methods opposes the biomedical approach to birth and instead aligns with the enduring midwifery philosophy of trusting the woman and her body^6^. Methods of NPPM include relaxation, active birth, water immersion, heat, massage, acupuncture, aromatherapy, a transcutaneous electrical nerve stimulation (TENS) machine and sterile water injections (SWI)^7^. Non-pharmacological pain management focusses on the body’s normal functions to leave birth processes undisturbed^6^. By doing so, the woman’s experience can be transformed from one of enduring pain to functional discomfort^8^. Labor pain management is a central concern and source of anxiety for expectant mothers, making it a challenge in midwifery care^1^. Because midwives have the opportunity to build rapport and trust with women throughout their labor, midwives play a central role in decision-making surrounding methods of pain management during labor^8^. By offering and encouraging the use of methods of NPPM strategies, midwives are able to promote and build a positive experience of childbirth^8^.

Pharmacological pain management has been shown to carry associated risks such as perceived lack of maternal control, delayed onset of the second stage of labor and increased further interventions such as instrumental birth^9^. Despite the associated risks with pharmacological methods, it appears the risk-to-benefit ratio of non-pharmacological and pharmacological management options are often overlooked by the woman whose priorities lie with the efficacy of pharmacological pain management techniques^9^. Statistical data show a 2% increase in the use of pharmacological methods of pain management and an 8.2% increase in the use of regional analgesia in Australia between 2011 and 2017^4,10^. These statistics are demonstrative of the increasing dependence women place on pharmacological pain management, prompting investigation into the cause of recent increases^4,10^.

One factor contributing towards the increased use of pharmacological pain management is the shifting paradigm of midwifery^11,12^. Women have different attitudes towards childbirth and these attitudes are influenced by the societal culture of birth^11^. In general, there has been a paradigm shift towards an obstetric-dominant and medicalized approach focused on risk management^12^. This has seen increased use of medical interventions such as electronic fetal monitoring systems, induction of labor and regional analgesia^11^. Although these advancements have seen a reduction in maternal and fetal mortality rates, they have also increased surveillance of not only the mother and baby, but the process of labor itself^12^. This risk-averse medical model is leading to a culture shift in women’s attitudes and expectations of birth such that women are losing confidence in their ability to give birth and to cope with labor pain^11^.

There are a variety of barriers preventing midwives from implementing NPPM^13^. Many midwives do not believe NPPM methods work in relieving pain compared to pharmacological approaches^5^. Additionally, midwives perceive non-pharmacological approaches as time-consuming and due to excessive workloads, insufficient staffing and clinical time constraints, do not feel time allows for NPPM methods^14^. A third concern is environmental barriers, such as inappropriate design and layout of birthing rooms^12,15^. Literature and clinical practice suggest there lies an area for inquiry surrounding the barriers to using NPPM in labor^5^. This review aims to provide a synthesis of evidence pertaining to midwives’ beliefs of and attitudes towards the barriers to offering NPPM as an initial option for laboring women.

## METHODS

### Aim

This review of the literature aimed to provide a synthesis of the evidence pertaining to midwives’ beliefs and attitudes towards the barriers to offering NPPM as an initial option for laboring women.

### Search strategy

An electronic database search was conducted using PubMed, Cumulative Index of Nursing and Allied Health Literature (CINAHL), Excerpta Medica Database, and Joanna Briggs Institute. Key search terms included: midwifery, midwife, barrier, obstacle, pain relief, pain management, analgesia and labor. Synonyms and truncations of these keywords were used, and database-specific medical subject headings (MeSH) were also included. Included studies had to be primary research that focused on midwives’, nurses’ or nurse/midwives’ perceptions of barriers^16^. Based on the current midwifery model internationally, both nurses and midwives work within the birth setting; as such, the population as defined in the inclusion and exclusion criteria included nurses, midwives or nurse/midwives that cared for women in labor^17^. Included studies had to be published between 2010 and 2020, written in English, available to be read in full, sourced from peer reviewed journals and be evaluated as moderate or high quality according to the CASP checklists^18-20^. The inclusion and exclusion criteria are detailed in Table 1.

**Table 1:** Inclusion and exclusion criteria for inclusion in the review of the literature

*Item*	*Inclusion criteria*	*Exclusion criteria*
Population	Midwives, nurses or nurse/midwives caring for women in labor	Midwives, nurses, or nurse/midwives not caring for women in labor
Source	Published in peer reviewed journals	Not published in peer reviewed journals
Publication dates	Published between 2010 and 2020	Published prior to 2010
Language	Published in English	Published in languages other than English
Availability	Full text available	Full text unavailable
Study design	Primary research that focuses on midwives’, nurses’ or nurse/midwives’ perceptions of barriers	Secondary research
Quality	Studies evaluated as moderate or high quality according to the CASP checklists	Studies evaluated as low quality according to the CASP checklists

CASP: Critical Appraisal Skills Programme.

NPPM methods include an application of a heat source, hydrotherapy, acupressure, acupuncture, hypnosis, relaxation, massage, yoga, TENS, aromatherapy, SWI, and birth balls^21^. Some studies discussed the barriers to NPPM in general by discussing many different types of pain management^5,12,14,15,22-24^. While other studies focused on the barriers to the use of one specific method of NPPM^25-30^. As both approaches fulfilled the aim of discussing the barriers to the use of NPPM, studies with both approaches were included.

**Figure 1:**
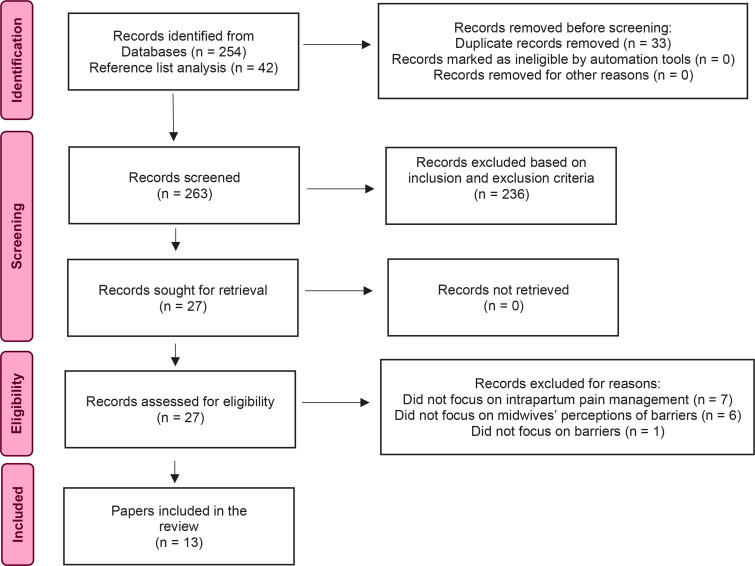
PRISMA flowchart demonstrating the search and screening strategy

The literature search identified 254 studies. References from all screened studies were assessed for any additional studies that were included (n=42) that met the inclusion criteria, and these studies then underwent the same screening process. Study duplicates were removed (n=33). Study abstracts (n=263) were read in full and screened against the inclusion and exclusion criteria. Twenty-seven studies met the inclusion criteria and 236 studies did not meet the inclusion criteria. The included studies (n=27), identified by the lead researcher, were then reviewed by two other midwifery researchers to assess for relevance and level of evidence. The three researchers discussed their findings, and a further 14 studies were excluded based on the exclusion and inclusion criteria. Reasons for exclusion were: the study did not discuss intrapartum pain management (n=7), the study did not focus on midwives’ perceptions of barriers (n=6), or the study did not focus on barriers (n=1). A final number of 13 studies underwent qualitative synthesis. The search process is outlined in the PRISMA flowchart (Figure 1)^31^. A PRISMA checklist is given in the Supplementary file.

### Data quality appraisal and synthesis

Prior to inclusion in the review of the literature, the quality of each study was assessed by three authors independently using the CASP checklists (Supplementary file, Appendices A and B)^32,33^. All authors consulted their findings with each other and agreement on the final studies for inclusion was reached. The authors defined moderate and high methodological quality as meeting 60–80% and 90–100% of the CASP checklist criteria, respectively^32-34^. The minimum percentage threshold for inclusion in the review of the literature was decided to be 60% of the criteria^34^. The quality of the review strategy was assessed using the AMSTAR 2 checklist of critical appraisal (Supplementary file, Appendix C) and was found to be moderate^35^. Once the 13 studies were identified, each underwent a five-step process of thematic development and synthesis based on the thematic analysis guide of Kiger and Varpio^36^. The five steps were:

1. Familiarized self with the data: repeated and active reading through of the data;
2. Generated initial codes: identified potential data items of interest, defined coding framework and made notes on potential patterns of data;
3. Searched for themes: examined the collated data to identify potential themes of broader significance;
4. Reviewed themes for proper fit; and
5. Defined and named themes.

## RESULTS

**Figure 2:**
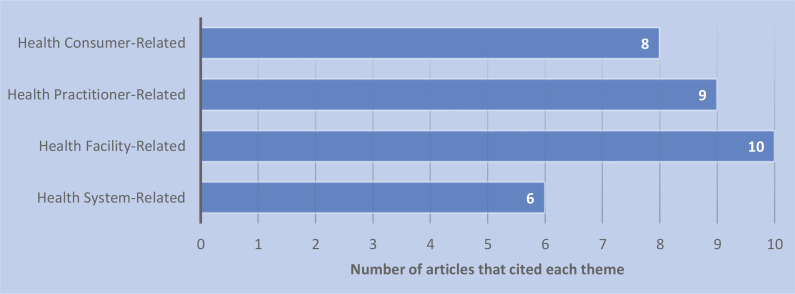
Total weight ranking of all the themes from the review of the literature

**Figure 3:**
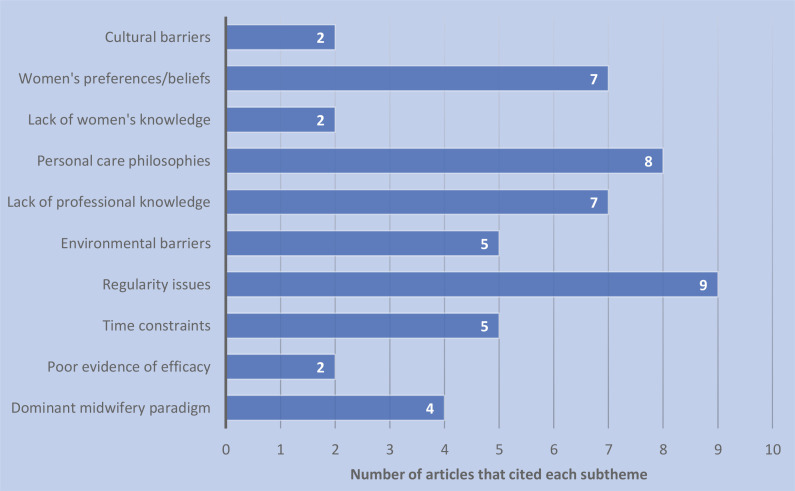
Total weight ranking of all the subthemes from the review of the literature

A summary of the included studies in the review of the literature can be seen in Table 2. The two qualitative approaches used were descriptive (n=6) and cross-sectional (n=4). The remaining studies were either a literature review (n=2) or used a mixed methods approach (n=1). The studies were conducted in Australia (n=3), the United States of America (n=2), Africa (n=2), Japan (n=1), the Netherlands (n=1), Brazil (n=1), Egypt (n=1) and Saudi Arabia (n=1). One cross-sectional study was conducted internationally (n=1). After the 13 studies underwent thematic development and synthesis, four main themes emerged: health system-related barriers, health facility-related barriers, health practitioner-related barriers and health consumer-related barriers. Beneath these four themes ten subthemes were also identified (Table 3). The respective weights of the themes and subthemes were calculated to indicate which barriers were of most clinical significance. These calculations found that regularity issues, personal care philosophies, lack of professional knowledge and women’s preferences/beliefs were the four most-cited barriers in the reviewed literature. A graphical representation of the weight rankings of the themes and subthemes can be seen in Figures 2 and 3, respectively.

**Table 2:** Included studies in the review of the literature

*Author (Year)*	*Study design*	*Setting*	*Sample size*	*Synopsis of results*	*CASP Checklist (%)*
Almushait and Ghani^22^ (2014)	Cross-sectional study	Abha Maternity Hospital, Saudi Arabia	88 registered maternity nurses and doctors	There are many barriers preventing nonpharmacological pain therapies from being used related to hospital regulations and policies.	Qualitative Checklist (100%)
Barrett and Stark^15^ (2010)	Secondary data analysis	United States of America	64 midwives and nurses	The birth environment may influence the intrapartum care given.	Systematic Review Checklist (100%)
Behruzi et al.^12^ (2010)	Qualitative field research	9 birthing centers or hospitals in Japan	44 midwives	There are many barriers to achieving humanized birth, including the cultural values and beliefs of women in Japan.	Qualitative Checklist (100%)
Boateng et al.^5^ (2019)	Qualitative: descriptive phenomenological	Two large hospitals in Ghana	15 midwives and nurses	Numerous barriers prevent utilization of non-pharmacological methods of pain management.	Qualitative Checklist (100%)
Cooper et al.^25^ (2018)	Mixed methods study	Australia	234 midwives	Policy and guideline documents limited midwives’ abilities to facilitate water immersion in labor.	Qualitative Checklist (100%)
Klomp et al.^14^ (2016)	Qualitative: descriptive focus-group study	23 midwifery practices in the Netherlands	23 midwives	There exists a conflict between midwives’ professional attitude towards normal birth and labor pain and the paradigm towards pharmacological management.	Qualitative Checklist (100%)
Lee et al.^26^ (2019)	Cross-sectional study	Royal College of Midwives’ Facebook page and Twitter account	398 midwives	Policies, lack of information and a lack of training are restricting the use of sterile water injections.	Qualitative Checklist (100%)
Lee et al.^27^ (2017)	Qualitative sub-study of a randomized controlled trial	Australia	11 midwives from 2 metropolitan maternity units	Midwives are challenged by the dilemma of inflicting pain to relieve pain.	Qualitative Checklist (100%)
Lee et al.^28^ (2012)	Cross-sectional study	Australia	970 midwives from the Australian College of Midwives	There exists a need for increased access to information and workshops on SWI to increase use in the clinical area.	Qualitative Checklist (100%)
Ramasamy et al.^23^ (2018)	Non-experimental cross-sectional descriptive study	Two referral and teaching hospitals in Kenya	286 labor nurses, midwives and midwifery students	The main barriers to providing non-pharmacological methods of pain management in labor were lack of time, lack of knowledge and women’s unwillingness.	Qualitative Checklist (90%)
Stark and Miller^29^ (2009)	Comparative descriptive survey	United States of America	401 intrapartum nurses	Birthing unit-specific barriers influence the perception of barriers to the use of hydrotherapy in labor.	Qualitative Checklist (90%)
Vargens et al.^24^ (2013)	Systematic literature review	Brazil	21 articles	Theoretical foundations and strategies need to improve to establish humanized care.	Systematic Review Checklist (100%)
Youness and Moustafa^30^ (2012)	Qualitative: descriptive	Tertiary hospital in Egypt	120 obstetric nurses	Providing hydrotherapy requires a supportive environment, adequate staffing, applied policies and collaborative relationships among the healthcare team.	Qualitative Checklist (100%)

**Table 3:** Summary of key themes and subthemes

*Themes*	*Subthemes*
Health system-related barriers	Dominant midwifery paradigm
	Poor evidence of efficacy
Health facility-related barriers	Time constraints
	Regulatory policy issues
	Environmental barriers
Health practitioner-related barriers	Lack of professional knowledge
	Personal care philosophies
Health consumer-related barriers	Lack of women’s knowledge
	Women’s preferences/beliefs
	Cultural barriers

### Health system-related barriers

The first theme of barriers to the initial administration of NPPM methods by midwives related to the wider healthcare system. This theme related to the overall structure of the healthcare and midwifery settings and how these structural elements shape the midwifery approach and contribute to the foundations of midwifery practice guidelines^22^. The two central barriers identified as subthemes in the synthesis of the studies in this theme included: the dominant midwifery paradigm and poor evidence of efficacy.

*Dominant midwifery paradigm*

The dominant midwifery paradigm related to the dominant values and approaches within the field of midwifery^37^. Four studies identified the current shift towards the biomedical model of care as a barrier in the implementation of NPPM in labor^14,15,24,29^. The interventionalist biomedical model of birth care is influencing the common perception of pain management towards pharmacological approaches for both midwives and women, to the point where pharmacological pain management is gaining a sense of normality^24^. Klomp et al.^14^ identified that women requested medical intervention for pain earlier than they did ten years ago, and some requested intervention before labor had even commenced. Hastening, controlling and mechanized birth has existed for long enough to influence the midwifery environment globally such that midwives who are used to the environment of intervention are less likely to use labor support techniques such as NPPM methods^15^. Midwives are finding it difficult to enact their ideal of keeping birth normal because this biomedical approach to birth care has caused conflicts between the fundamental paradigms of midwives and those of their medical colleagues^14^. Stark and Miller^29^ in their comparative descriptive study specifically investigated the barriers to the use of hydrotherapy in labor. These authors found that when midwives were the primary intrapartum carer, they perceived fewer barriers to hydrotherapy in comparison to situations where physicians were the primary carer^29^.

*Poor evidence of efficacy*

In the synthesized studies, midwives reported that poor evidence of efficacy of NPPM methods was a common barrier to their implementation^23,26^. Ramasamy et al.^23^, in their cross-sectional descriptive study, found that 91% of midwives viewed poor evidence of efficacy as a barrier to the implementation of NPPM. Another cross-sectional study by Lee et al.^26^ investigated the barriers to sterile water injections (SWI) in particular and found that 13.3% of midwives reported a lack of supportive evidence discouraged their use of SWI in clinical practice. A further 14.4% of midwives believed this lack of evidence contributed to a lack of confidence when using the procedure as they themselves were unsure whether the procedure was safe^26^.

### Health facility-related barriers

The second theme of identified barriers to the use of NPPM related more specifically to health facilities, and the three central barriers identified as subthemes included: time constraints (including human resourcing), regulatory policy issues and environmental barriers.

*Time constraints (including human resourcing)*

Issues regarding human resources and staffing were cited in the synthesized studies as a key barrier to providing NPPM methods^22,23,25,30^. Three studies found that 41%^30^, 63.7%^22^ and 92.9%^23^ of midwives believed inadequate staffing was a barrier to the use of non-pharmacological techniques. Cooper et al.^25^, in their mixed methods study, investigated the barriers to hydrotherapy in labor and found that 33.5% and 35.4% of midwives identified limited availability of accredited staff for water immersion as a major barrier and a moderate barrier, respectively. Inadequate staffing leads to increasingly overwhelming responsibilities for midwives and nurses in the clinical setting^5^. These time constraints were identified as a barrier to the use of NPPM for 95.5%^23^ and 76.1%^22^ of midwives in two separate studies. In some situations, midwives believed that the time constraints limited the space for conversation regarding anything beyond standard practice, limiting their ability to engage with women in relation to working with labor pain^14^. Youness and Moustafa^30^ found 48% of intrapartum nurses identified the increased effort required to administer non-pharmacological interventions as a barrier to its use. Midwives believed NPPM methods are more time-consuming as they require continuous support and due to the lack of staff and time constraints, midwives believe they do not have the time to properly and safely implement non-pharmacological techniques^5^.

*Regulatory policy issues*

Limiting hospital policy was identified as a barrier in 9 of the 13 studies in the reviewed literature^12,14,22-26,28,30^. Youness and Moustafa^30^ found 100% of intrapartum nurses considered hospital policy as a barrier to the use of NPPM in labor, with two other studies also finding that 87.5%^22^ and 82.2%^23^ of midwives identify this is as an issue. Policy and regulations surrounding intrapartum pain management reflect the shifting paradigm of midwifery^14^. Klomp et al.^14^, in their qualitative focus group study of Dutch midwives, found that the implementation of a guideline entitled ‘Pharmacological Pain Relief’ in the Netherlands changed women’s attitudes towards labor pain, and in turn changed the way midwives supported women towards more pharmacological methods. These researchers noted that there was an apparent shift towards a more pharmacologically orientated approach to pain management in the Netherlands after this guideline was introduced^14^.

Findings of the review of the literature showed that most hospital policies used to underpin the care of laboring women did not support the use of NPPM^12,24-26,28^. In a study by Behruzi et al.^12^ investigating the role of companions as a natural method of pain management, midwives identified that hospital policies restricted the number of companions allowed during labor and birth, and this was perceived to impact women’s mentality to pain and overall comfort^12^. The study by Cooper et al.^25^ focused solely on water immersion in labor, and these researchers found that 12.7% of midwives believed that the policies and guidelines did not facilitate the practice of water immersion for labor and birth. As a result, 27.5% of midwives believed these regulatory policy issues restricted women’s choice and autonomy when it came to the use of water immersion^25^. Two studies, investigating the barriers towards the implementation of SWI as a method of NPPM in labor, found that 74%^26^ and 57.5%^28^ of midwives believed the lack of supporting policy or guideline was the main barrier to its implementation. Interestingly, these studies also found that 39.6%^28^ and 17.8%^26^ of midwives stated that no policy or guideline was available, and therefore there was a lack of support from the institution for its use.

*Environmental barriers*

Environmental barriers such as limited resources and architectural limitations were also identified by midwives as barriers to the use of NPPM methods. Midwives suggested that the limited number of rocking chairs, birthing balls, whirlpools and showers was a barrier to the use of NPPM^15^. Small or congested room restrictions that provided limited space for walking, mobilization and position changes during labor was another reported barrier^12^. Three studies^25,29,30^ specifically addressed environmental barriers in relation to water immersion in labor. One study found 52%^30^ of nurses believed that a lack of resources including birthing pools limited the use of intrapartum hydrotherapy. Two main infrastructure-related limitations included poor availability of baths and birthing pools, and limited lifting equipment for evacuation^15,25^.

### Health practitioner-related barriers

The third theme of identified barriers to the use of NPPM related to health practitioners, and the two central barriers identified as subthemes included: lack of professional knowledge and personal care philosophies^5,15,22,23,25-28,30^.

*Lack of professional knowledge*

A lack of professional knowledge surrounding NPPM methods was identified as a barrier by 93.2%^23^ and 81.8%^22^ of midwives in two separate studies. The study of Barrett and Stark^15^ identified that the number of years of clinical experience as the birth care provider was a contributing factor to this barrier. Midwives with less experience felt less confident with labor support including NPPM, and instead were more comfortable with technology-related tasks and interventions^15^. Specifically relating to water immersion, 26.7% of midwives in the study of Youness and Moustafa^30^ identified poor knowledge about hydrotherapy as a barrier to its use. The study by Cooper et al.^25^ found that 56.5% of midwives had not been taught about water immersion for labor as part of their midwifery education and 37% of midwives knew only a small amount of information. Two studies investigated midwives’ knowledge surrounding SWI and found that 90%^28^ and 86%^26^ of midwives believed they required more information about the procedure. Two barriers were identified for midwives who were not using SWI, and these included limited access to workshops and education (14.4% of midwives)^26^ and poor access to education materials (10.9% of midwives)^28^. One midwife stated^28^:

*‘Local competency requirements are prohibitive, and for MGP midwives it is almost impossible to get an approved clinician to supervise to deem you competent.’*

*Personal care philosophies*

Another identified barrier to the use of NPPM in labor was the perceptions and beliefs of midwives and nurses that non-pharmacological interventions do not relieve pain^5^. Midwife and doctor unwillingness towards use of NPPM was cited as a barrier for 93.6%^23^ and 67.1%^22^ of midwives in two separate studies. Cooper et al.^25^ also noted that medical and obstetric personnel aversion of NPPM methods contributed to their underutilization. Lee et al.^28^ found that resistance from medical and midwifery colleagues towards SWI use was a barrier for 25% of midwives. One midwife from the qualitative analysis of this study stated^28^:

*‘I see it as an intervention and prefer not to use needles at all in my practice.’*

Three studies^26-28^ specifically addressed the personal care philosophies of midwives towards SWI. Many midwives reported that their underlying philosophic belief that SWI were invasive prevented their use in clinical practice^27^. Midwives saw their role in pain management as a supportive one, and therefore did not believe they should cause more pain in any way^27^. For midwives who had never used SWI, the pain it caused was a barrier to its use^27^. Midwives’ past experiences of administering SWI also played a role in their willingness to suggest and use SWI, as midwives believed that negative experiences of pain associated with SWI administration influenced their decision and likelihood to use it again^27^.

### Health consumer-related barriers

The fourth theme of identified barriers to the use of NPPM related to the women themselves, and the three central barriers identified as subthemes included: a lack of women’s knowledge, women’s preferences/beliefs and cultural barriers^5,12,14,15,22,23,27,30^.

*Lack of women’s knowledge*

Two studies identified women’s knowledge as a barrier to the implementation of NPPM in labor^14,15^. Contributing to this was the perceived diminished amount of antenatal education provided to women as well as the overall portrayal of labor and childbirth in the media^14^. The media surrounding pain management seem to portray the pain relief view where women are made to believe that they do not want to feel pain and should use pain medications as a substitute for support^14^. The lack of education about the use of pharmacological pain management in the antenatal period could lead to women’s increased reliance on media for knowledge acquisition, and this is leading to a misrepresentation of labor pain, an increase in women’s fear of labor and a reliance on pharmacological methods of pain management^14^. Because of the media’s influence, midwives also believed that they were no longer able to use their midwifery knowledge and education to influence a woman’s decisions regarding pain management as there seems to be a lowering threshold for the use of pharmacological pain management among women^14^.

*Women’s preferences/beliefs*

Another barrier to using NPPM identified through the review of the literature was that women had strong beliefs of pharmacological analgesia^22,23^. Women believed that NPPM methods were not concrete, meaning these methods had doubtful efficacy and often did not eradicate pain^22,23^. Two studies found that 93.6%^23^ and 82.9%^22^ of midwives identified that the woman’s strong belief of pharmacological analgesia was a barrier to the use of NPPM. These two studies found that 91%^23^ and 81.8%^22^ of these women believed that NPPM options were not as concrete as pharmacological options, and therefore were not considered reliable enough to try.

The synthesized literature in this review found that women and families had their minds made up regarding methods of pain management prior to the establishment of labor, including plans to use epidural analgesia, and this was another identified barrier^15^. As such, women were unwilling to try NPPM methods^5^. Two studies found that 94.4%^23^ and 85.2%^22^ of midwives identified women’s unwillingness to trying NPPM methods as a barrier to their use. Klomp et al.^14^ found that midwives believed they had limited influence on pain management approaches because of the individual woman’s perspectives and predetermined approaches. Some women do not accept the supporting role of a midwife because their personal philosophy towards pain may differ from that of the midwife, and as such midwives felt unable to influence the process of labor pain management^15^. Midwives also found the subjective nature of pain complicated their individual recommendations surrounding pain management^27^.

*Cultural barriers*

Cultural influences are cited as a potential barrier to the use of NPPM methods in two studies^12,14^. Midwives noted that individual cultures and regions of origin have specific beliefs about labor pain and different approaches to pain management^14^. One qualitative field research study focused on the Japanese culture whereby women commonly lack the confidence to make decisions in hospitals based on their culture of obedience^12^. As such, Japanese women are more likely to follow the recommendations of the medical staff without voicing their preferences or concerns^12^. The shift towards a biomedical model of care, which is occurring internationally, may increase the chance of pharmacological intervention use for these women^12^. Klomp et al.^14^ specifically touched on the cultural beliefs about labor pain management of women from Africa and the Netherlands. These specific cultural beliefs can make it more difficult for midwives to appropriately coordinate pain management use and help women manage their labor pain because they are required to possess a diverse range of culturally appropriate support skills. Two midwives stated^14^:

*‘We also have a very special group of mostly parous women from Africa. These women have given birth before, in Africa, without pain relief. They just give birth without [pain medication], they don't even question it. They do not see this as an issue, it is just something you do.’*

*‘In our region (the religious region of the Netherlands), people believe what is written in the Bible: “Thou shalt give birth in grief” … For this reason, in our practice, many women do not want to use pain relief, and they wonder “is it permissible to use labor pain medication?”.’*

## DISCUSSION

The results of this review of the literature indicate that midwives and nurses who care for laboring women believe there are significant barriers that prevent their utilization of NPPM as an initial option for laboring women. In the studies reviewed, the most cited barrier identified by midwives was that maternity policy and guidelines did not always support the use of NPPM methods in labor particularly surrounding intrapartum energy-level support, companionship, water immersion in labor and SWI^14,24-26,28^. Midwives perceived these regulatory policy restrictions as a lack of support from the institution to employ NPPM methods, and thus believed that the dominant biomedical model of birth care is influencing both midwifery practice and policy generation^26^. Lacking or restrictive policies decrease the opportunities for use of NPPM in the clinical setting, and also limit the trust staff and women place in these methods^12,14,22-26,28,30^. It is important that policies surrounding pain management are flexible and incorporate patient experiences because of the subjective nature of pain and unpredictability of pain progression^38,39^. Effective policy generation will minimize barriers and promote the use of NPPM methods^39^.

Although midwives and nurses are focused on upholding the midwifery role of keeping birth normal, the dominant paradigm of intervention and the biomedical model of care that currently exists within the midwifery landscape is a barrier preventing the use of NPPM methods^14,29^. This dominant biomedical model of labor care is changing the common perception of pain management towards using pharmacological pain management for both women and midwives^24^. Caring for women in labor is currently shaped by medical technology to the point where most elements of practice, including pain management, are focused on the biomedical model of obstetric care^40^. The biomedical model of midwifery and birth care has mechanized birth to the point where all four levels of influence have been affected: the wider healthcare system, individual healthcare facilities, midwives, and women^13^.

There is limited and inconsistent research surrounding the safety and efficacy of NPPM methods to the point where midwives believe this limits their willingness to implement such methods^23,26^. The identification of adverse events in epidemiological research is more effectively determined through large observational cohort data, however, this form of research is lacking in the field of NPPM^41^. There is a need for evidence-based care when assessing the appropriateness of NPPM, as without this, midwives are left to rely on experiential and intuitive understanding^42^.

The review demonstrated that insufficient staffing served as a barrier to the utilization of NPPM methods in labor^22,23,25,30^. The nature of NPPM methods compounds this issue as they can be time-consuming to apply and can require more than one midwife to facilitate^5,30^. Insufficient staffing contributes to unrealistic workloads and increased stress among midwives and labor care providers, and these factors ultimately lead to poor work efficiency^43^. There is a need for review of human resources and staffing requirements to support midwives to be in a position to work in partnership with women during labor^5^.

The review revealed that environmental barriers also prevented midwives and nurses from implementing NPPM in labor^12,15,25,30^. The nature of the birthing environment has a significant impact on the methods of pain management women utilize, with evidence showing that greater mobility and longer periods of active labor result in a significantly decreased probability of using pharmacological pain management^44^. In the studies reviewed, midwives identified that the inappropriate design and layout of labor rooms in addition to limited availability of birth balls, rocking chairs, showers and baths restricted the methods of NPPM they could offer women^12,15,25,30^. There is a need for increased resource availability and appropriate labor room design to promote and support the use of mobilization, active methods of birth and other non-pharmacological approaches in labor^44^.

A lack of professional midwifery knowledge was cited by midwives as a significant barrier to the use of NPPM in labor^15,22,23,25,26,28,30^. The three main factors that contributed to insufficient knowledge was clinical inexperience of the midwife, an inability to access educational workshops or resource material, and an inability to access approved clinicians to gain competency in certain methods of NPPM^15,25,26,28^. Insufficient professional knowledge of birth care providers can lead to misconceptions regarding the efficacy of NPPM which in itself is a barrier to its use^45^. Midwives’ poor knowledge also impairs their ability to use methods of NPPM in the correct clinical context^45^. There is a link between professional knowledge, the amount of hands-on intrapartum experience and increasing confidence, highlighting the importance of knowledge for clinical confidence and competency in midwives^46^.

An important method of health promotion when it comes to NPPM is providing education to midwives, particularly surrounding the physiology of labor pain, the available methods of NPPM and common methods of pain assessments^47^. There is also value in educating midwives on the adverse effects of pharmacological methods of pain management to increase their confidence in promoting methods of NPPM^48^. Increasing education, support and confidence surrounding methods of NPPM will help expand their overall use in clinical practice^46^.

The reviewed literature indicates that the use of NPPM is limited by personal care philosophies of both midwives and medical staff^5,22,23,25-28,30^. The review found midwives were challenged by differences in professional ideology towards NPPM between themselves and other midwifery or medical colleagues^5,25^. Some colleagues did not accept NPPM as ‘real’ labor pain management and would avert conversations surrounding these forms of pain management^5,25^. Fundamental paradigm clashes within the maternity sector create a ‘tug-of-war’ between approaches of NPPM and conventional medicine to the point where midwives feel influenced by the opinions of other professionals to conform to the pharmacologically focused approach to intrapartum pain management^42^.

The philosophical approach underpinning midwifery is one that prioritizes the personal beliefs and preferences of the woman above that of the midwife or institution, and this is no different when it comes to pain management^49^. Midwives in six studies indicated that women’s birth preferences and general beliefs about labor and labor pain affected their provision of NPPM^5,14,15,22,23,27^. Women had strong beliefs of pharmacological analgesia and doubted the efficacy of non-pharmacological methods to the point where some had their minds made up about their desires for pain management before labor commenced^14,15^. In these instances, midwives found it difficult to use their clinical knowledge and expertise to influence labor pain management^14^. The literature is clear about the advantages of creating an individualized environment that is conducive to each woman’s preferences, as it can effectively reduce painful sensations during labor^49^.

In one study, midwives expressed concern surrounding the impact of the media on women’s perceptions of birth and labor pain^14^. The media are increasingly favoring the view of childbirth as risky and painful and generally support the ‘pain relief’ approach to childbirth, which has been found to contribute to misconceptions surrounding the necessity of pharmacological pain management^50^. The media are an influencing factor that shapes women’s ideas and beliefs surrounding labor and pain management and therefore are a factor lowering the threshold for using pharmacological pain management among women^14,50,51^. Despite efforts to build trust with women, midwives feel limited in their ability to influence pain management techniques because of an overall lack of understanding of NPPM^14^.

Another factor that influences women’s approach to pain management is specific cultural beliefs and practices regarding labor pain management^15^. Cultural considerations in the humanization of birth is a focus for birth care, and research supports the belief that cultural components of a woman’s milieu influence her choices regarding birth practices, including pain management^12^. In Australia, a priority area is the provision of culturally competent maternity care to Aboriginal and Torres Strait Islander women^52^. Providing culturally sensitive and respectful maternity care and bettering communication for Indigenous women and their families have been found to increase satisfaction and improve pain management^53^.

Patient education is a key method of health promotion that can be used to address this lack of understanding and awareness when it comes to pain management^47^. There are three important goals of patient education in the realm of intrapartum pain management. The first, and one that underpins all other elements of pain management education, is to improve women’s understanding of pain and how labor pain and labor pain management differ from other forms of pain^8^. Developing this understanding will assist women to make informed decisions surrounding their pain management^8^. The second important area of patient education is surrounding the different methods of NPPM available to women, including the risks and benefits of each^8^. Finally, it is important to acknowledge and educate women on the alternative, being pharmacological pain relief, and particularly the adverse effects of pharmacological methods of pain management^8^. Providing robust discussions surrounding these three topics throughout the antenatal period is an important element of antenatal care to promote the use of methods of NPPM^8^.

### Limitations

There were two main limitations of this review of the literature. Firstly, a review of the literature does not apply the same rigor in the methodology when compared to a systematic review. Therefore, it is possible that some articles were missed. Secondly, the literature search covered up to 2020. During the time between completion of the literature search and publication, a new study may have been published that was applicable to this review of the literature. To mitigate these limitations, a recent search of the literature was conducted in December 2021 and no further articles were found.

## CONCLUSIONS

In order to enhance the quality of care provided to women, midwives should be aware of the barriers that prevent or delay the utilization of non-pharmacological methods of pain management in labor. The findings of this review of the literature demonstrated that four main barriers exist: health system-related, health facility-related, health practitioner-related and health consumer-related barriers. These findings can be used as a platform to inform further research into this topic. By developing the evidence base surrounding NPPM, it is hoped that women’s dependence on pharmacological pain management will decrease and the approach to birth care can shift away from a biomedical one to a model that mirrors the enduring midwifery philosophy of trusting the woman, trusting her body and being ‘with woman’.

## Data Availability

Data sharing is not applicable to this article as no new data were created.
